# Validation and the associated factors of the Malay version of systemic lupus erythematosus-specific health-related quality of life questionnaires (SLEQoL and LupusQoL)

**DOI:** 10.1371/journal.pone.0285461

**Published:** 2023-05-15

**Authors:** Nur Aqeelah Ahmad Pouzi, Syahrul Sazliyana Shaharir, Azmi Mohd Tamil, Ruslinda Mustafar, Suhaida Ahmad Maulana, Eashwary Mageswaren, Wan Syamimee Wan Ghazali

**Affiliations:** 1 Department of Internal Medicine, Faculty of Medicine, Universiti Kebangsaan Malaysia, Jalan Yaacob Latiff, Cheras, Kuala Lumpur, Malaysia; 2 Department of Public Health, Faculty of Medicine, Universiti Kebangsaan Malaysia, Jalan Yaacob Latiff, Cheras, Kuala Lumpur, Malaysia; 3 Medical Department, Hospital Tengku Ampuan Rahimah, Klang, Selangor, Malaysia; 4 Department of Internal Medicine, School of Medical Sciences, Health Campus, Universiti Sains Malaysia, Kubang Kerian, Kelantan, Malaysia; Universiti Sains Malaysia, MALAYSIA

## Abstract

**Objectives:**

To assess the reliability and validity of two disease-specific questionnaires that assess the quality of life (QoL) among patients with Systemic Lupus Erythematosus (SLE); SLEQoL and LupusQoL in Malay language. This study also identified the factors affecting each domain of the questionnaires.

**Methods:**

This cross-sectional study was conducted from June 2021 until April 2022, and SLE patients were recruited to complete the SLEQoL, LupusQoL and Short Form Health Survey (SF-36) in Malay language. Disease activity were recorded using the modified SLE Disease Activity Index (M- SLEDAI) and British Isles Lupus Assessment Group 2004 (BILAG-2004) index. Presence of organ damage was determined using the SLICC Damage index. Cronbach’s alpha was calculated to determine internal consistency while exploratory factor analysis was done to determine the construct validity. Concurrent validity was evaluated using correlation with SF-36. Multiple linear regression analysis was deployed to determine the factors affecting each domain of SLEQoL and LupusQoL.

**Results:**

A total of 125 subjects were recruited. The Cronbach’s α value for the Malay-SLEQoL (M-SLEQoL) and Malay-LupusQOL (M-LupusQoL) was 0.890 and 0.944 respectively. Exploratory factor analysis found formation of similar number of components with the original version of questionnaires and all items have good factor loading of >0.4. Both instruments also had good concurrent validity with SF-36. M-SLEQoL had good correlations with BILAG-2004 and M-SLEDAI scores. Musculoskeletal (MSK) involvement was independently associated with lower M-SLEQoL in physical function, activity and symptom domains. Meanwhile, MSK and NPSLE were associated with fatigue in M-LupusQoL.

**Conclusion:**

Both M-SLEQoL and M-LupusQoL are reliable and valid as disease -specific QoL instruments for Malaysian patients. The M-Lupus QoL has better discriminative validity compared to the M-SLEQoL. SLE patients with MSK involvement are at risk of poor QoL in multiple domains including physical function, activity, symptoms and fatigue.

## Introduction

Systemic Lupus Erythematosus (SLE) is a complex multisystem autoimmune disease with a wide spectrum of the clinical phenotype. The course and outcomes of SLE are influenced by several factors including genetic, environmental and sociodemographic background [1]. Despite the improvement of survival due to the advances in the treatment of SLE, their quality of life (QoL) is negatively affected due to the substantial morbidity from the disease and the treatment itself. It has been demonstrated that QoL is reduced in patients with SLE compared with patients Sjögren’s syndrome and rheumatoid arthritis [2], and with the general population [3]. Recognizing the importance of measuring QoL in SLE, the Outcome Measures in Rheumatology (OMERACT) group [4] and various regulatory agencies including European Medicines Agencies (EMA) and the United State Food and Drugs Administration (U.S. FDA) recommend that all drugs trial should include patient-reported outcome (PRO) as one of the therapeutic outcomes [5, 6].

However, the PRO tools to assess QoL in SLE are mostly designed in English and hence mainly being validated among English-speaking population [7]. Currently, in Malaysia, there is lack of a valid and reliable PRO instrument for our Malaysian SLE patients. So far, only the generic Medical Outcomes Study Short-Form 36 (SF36 Malay version) questionnaire has been validated in Malaysia [8]. Employing the generic tool to assess QOL in SLE may not adequately capture symptoms or issues that are specific to the disease [9]. Therefore, there is an urgent need to validate the PRO tools among our Malaysian SLE population to adapt with the local linguistic and cultural differences. Validated PRO tool in different languages enable more participation of patients from all over the world in randomized controlled trials (RCTs) to prevent study bias by lack of recruitment of patients from the non-English speaking countries in clinical trials [10].

The most-widely used SLE disease-specific PRO tool is LupusQoL which was developed in the UK [11]. LupusQoL has been widely validated and adapted to other cohorts and has been translated into 77 languages for use in 51 countries. Itis more being widely used in randomized–controlled clinical trials (RCTs) [9]. SLEQoL was initially developed and validated in English language among Singaporean Chinese patients. This tool incorporates questions suitable for oriental cultures [12], and since Malaysia is a neighbour country of Singapore, the content may be more culturally adapted with our population. It consists of 40-items and it demonstrates better responsiveness to change than the SF-36. The Chinese version of SLEQoL has also been validated in Singapore [13] and in addition, it has been translated and validated in Thailand [14] and Egypt [15] using their native languages.

There are various factors that can affect the QoL in SLE patients [16] and literature on QoL among Malaysian SLE population is still sparse. This is likely due to no disease-specific tool that is available in Malay language. Hence, there is an urgent need to validate the QoL assessments tool in Malay language, to encourage clinician to use these tools to assess the QoL aspect of patients. The validation may also enable more Malay patients who are not proficient in English language to participate in the international RCTs. In addition, this will also encourage the clinician to use these validated instruments and thus will further enhance the holistic approach of the management of the SLE patients in Malaysia.

In this study, we aim to determine the validity and reliability of two disease-specific patient reported outcome (PRO) instruments; SLE Quality of Life (SLEQoL) and Lupus Quality of Life (LupusQoL) in Malay language among SLE patients in two major Rheumatology centres. In addition, this study also evaluates the correlations and associations between the PRO instruments with SF-36, socio-demographic characteristics, SLE disease activity, presence of co-morbidities and presence of organ damage.

## Materials and methods

### Patients

This study was a cross-sectional study conducted in Hospital Canselor Tuanku Muhriz (HCTM) Universiti Kebangsaan Malaysia and Hospital Tengku Ampuan Rahimah (HTAR) Klang from June 2021 until April 2022. Adult SLE patients (≥ 18 years old) who fulfilled the American College Rheumatology (ACR) 1997 classification criteria [17] and/or Systemic Lupus Erythematosus Collaborating Clinics (SLICC) 2012 classification criteria [18] were recruited. Patients that were included have to be proficient in spoken and written Malay language. Patients were recruited from the outpatient clinics and convenience sampling method was used to recruit patients.

Patients with documented severe cognitive impairment (dementia, Alzheimer’s disease), psychiatric illness, and permanent physical disability not related to SLE or had an overlap SLE with other connective tissue diseases were excluded. Written informed consent was obtained from all patients and this study obtained approval from the UKM Ethics Committee (JEP-2021-332) and National Medical Research Register (NMRR DI-21-02263-DCN).

#### Data collection

While SLE disease characteristics were majorly extracted from patients’ medical records i.e. age of onset, duration of disease, organ or system involvement, and co-morbidities (hypertension, diabetes mellitus, ischemic heart disease), some sociodemographic data was obtained (e.g. age, gender, ethnicity, level of education, and type of occupation) via self-reporting google form or during clinical interviews. Patients self-completed the Malay version of SLEQoL (M-SLEQoL), LupusQoL (M-LupusQoL) and SF-36 (M-SF36) using hardcopies or google form.

#### Sample size calculation

All of the questionnaires (SF-36, M-SLEQoL and M-LupusQoL) that were used in this study have more than 5 loadings per item and based on the highest loading per item in M-SLEQoL (40 items), the calculated sample size was 200 patients [19].

#### SLE disease activity and damage

SLE disease activity assessments were performed at study visit in all patients using the modified-Systemic Lupus Erythematosus Disease Activity Index (M-SLEDAI) and British Isles Lupus Assessment Group Index (BILAG 2004) score [20]. Modified-SLEDAI which omits the serology items (complement levels, anti-dsDNA antibodies) is a validated and reliable instrument for disease activity assessments [21]. Total BILAG 2004 index scores were calculated based on category i.e. A = 12, B = 8, C = 1 and D/E = 0 [22]. Presence of irreversible organ damage was determined using the Systemic Lupus International Collaborating Clinics/American College of Rheumatology (SLICC/ACR) Damage Index (SDI) [23]. Patients were divided into 2 groups: those with damage (SDI score ≥1) and those without damage (SDI score = 0). Both disease activity and organ damage assessments were verified by a Rheumatologist who was blinded to the cases.

### Validation of the Malay version of SLEQoL and LupusQoL (M-SLEQoL and M-LupusQoL)

The validation of the Malay version of SLEQoL and LupusQoL (M-SLEQoL and M-LupusQoL) and consisted of 5 stages:

1. Content, translation and face validity
2. Internal consistency reliability by determining the Cronbach’s-α [24].
3. Construct validity using exploratory factor analysis [25]
4. Concurrent validity with the generic tool Short Form Health Survey in Malay language (M-SF36)
5. Discriminant validity with disease activity and damage

#### Content, translation and face validity of the M-SLEQoL and M-LupusQoL

Both of the original version of SLEQoL and LupusQoL questionnaires are in English language and they have been translated to Malay language by the original authors according to the industrial standard. However, the Malay version of SLEQoL (M-SLEQoL) and LupusQoL (M-LupusQoL) have never been validated.

Content validity was performed by the expert panels of the researchers which consists of 2 rheumatologists, and an academician public health specialist experienced in the validation of instruments. They have reviewed the content and decided that all the items were relevant and important. The Item-content validity index (I-CVI) and scale content validity index of both questionnaires were 1 (S1 File). The expert panels have also reviewed the translated version of the questionnaires and found that they were similar as the original version.

Face validity was done through cognitive debriefing of the questionnaires in 10 healthy subjects and 10 SLE patients to determine items clarity and comprehensibility. They were asked for their views regarding the phrasing and content of the instruments. All subjects understood the translated version and did not encounter problems in answering the questionnaire. However, we did not record the subjects’ rates on the items in the questionnaires and hence the Item-face validity index (I-FVI) and (Scale-face validity index) S-FVI were not calculated.

We have obtained permission from the original authors for the validation process. Table 1 compares the domains, response format and score interpretation of SLEQol and LupusQoL questionnaires.

**Table 1:** Comparisons between Lupus QoL and SLE QoL.

Measure	Recall period	Domains	Response format	Range Of Scores	Score interpretation
Lupus QoL [11]	4 weeks	34 items grouped into 8 domains: Physical health, emotional health, body image, pain, planning, fatigue, intimate relationships, and burden to others	5 -point Likert scale	0–100	Higher score indicates better quality of life
SLE QoL [12]	1 week	40 items grouped into 6 domains: physical functioning, activities, symptoms, treatment, mood, and self-image	7-point Likert scale	40–280	Higher score indicates worse quality of life

Subsequently the Malay versions of both questionnaires (M-SLEQoL and M-LupusQoL) were used in SLE patients for further testing of their reliability and validity. The subjects also completed the Malay version of the Short Form Health Survey (M-SF36) which has been validated and extensively used in patients with chronic diseases including SLE in Malaysia [8, 26–29].

### Statistical analysis

Data were analysed using the IBMM SPSS software (version 26). Kolmogorov Smirnov test was performed to assess normality of the data and the results showed that all variables had p<0.001, indicating that data was not normally distributed. Therefore, non- parametric statistical analysis methods were used.

Cronbach’s-α was performed to test the internal consistency of the questionnaires and was considered good if > 0.7 [24]. To determine the construct validity, exploratory factor analysis (EFA) was conducted to extract the factor structure of the questionnaires [25]. Prior to EFA analysis, Kaiser-Meyer-Olkin (KMO) and Bartlett’s tests were done to ensure sampling adequacy and suitability. The sampling is adequate if the value of KMO test is more than 0.5 while for Bartlett’s test, the significant value of less than 0.05 is acceptable for further analysis. The six factors in M-SLEQoL and 8 factors in M-LupusQoL were computed based on the factor extracted by the original questionnaires. The factors were rotated with Varimax rotations so that each variables loads only on one factor/component. This was an orthogonal method that ensures that the factors are uncorrelated. Variables with factor loadings of 0.4 and above were acceptable and were considered stable [30].

Concurrent validity was determined by performing bivariate Spearman’s rho test with SF36 while discriminant validity with disease activity scores (M-SLEDAI and BILAG-2004) and damage index (SDI). Spearman’s correlation r_s_>0.7 is regarded as showing very strong positive correlation; r_s_ from 0.4 to 0.69 suggests strong positive correlation, r_s_ from 0.30 to 0.39 suggests moderate positive correlation, r_s_ from 0.20 to 0.29 suggests weak positive correlation, r_s_ from 0.19 to −0.19 suggests no or negligible correlation, r_s_ from −0.20 to −0.29 suggests weak negative correlation, r_s_ from −0.30 to −0.39 suggests moderate negative correlation, r_s_ from −0.40 to −0.69 suggests strong negative correlation and −0.70 or lower suggests very strong negative correlation.

To determine factors associated with QoL, Mann Whitney test and Kruskal Wallis test were conducted for 2 categorical and ≥ 3 categorical variables respectively and Spearman’s test for continuous data. Subsequently, multivariate linear regression analysis which included all variables with p value of <0.10 was performed to determine the independent predictors of each QoL domain. In all analyses, a P value (two-tailed) of less than 0.05 was considered statistically significant.

## Results

### Socio-demographics and disease characteristics

A total of 125 patients were recruited and this study cohort was comprised of predominantly females (92%, n = 115) and Malay ethnicity (83.2%, n = 104). Majority of the patients were female n = 115(92%) while only n = 10(8%) were male patients. Majority were in the low socioeconomic category (household income less than Malaysian Ringgit, MYR 4,849/month or USD1,018/months). Table 2 illustrates the baseline socio-demographics and disease characteristic of the SLE patients.

**Table 2:** Socio-demographics and disease characteristics of the SLE patients.

Characteristics	Frequency, n (%)
Age, years	36.4 ± 11.7
Disease duration, years	7.6 ± 6.8
Gender	
Female	115 (92%)
Male	10 (8%)
Race	
Malay	104 (83.2%)
Chinese	13 (10.4%)
Indian	6 (4.8%)
Others	2 (1.6%)
Religion	
Muslim	106 (84.8%)
Buddhist	10 (8%)
Hindu	4 (3.2%)
Christian	4 (3.2%)
Education level	
Higher education	70 (56%)
Secondary education	55 (44%)
Marital status	
Married	77 (61.6%)
Single	38 (30.4%)
Divorced	8 (6.4%)
Widow	2 (1.6%)
Employment	
Unemployed	45 (36%)
Employed	80 (64%)
Household Income	
Less than MYR 4,849 (low income)	100 (80%)
More than MYR 4,850	25 (20.0%)
System involvement	
Musculoskeletal	88 (70.4%)
Haematological	47 (37.6%)
Mucocutaneous	62 (29.6%)
Renal	37 (29.6%)
Neuropsychiatric lupus (NPSLE)	16 (12.8%)
Comorbidities	
Hypertension	28 (22.4%)
Dyslipidemia	15 (12%)
Diabetes mellitus	7 (5.6%)
Heart disease	6 (4.8%)
M-SLEDAI score	0 (0–19)
BILAG-2004 score	0 (0–60)
SDI damage index	
Presence of damage (SDI≥1)	23(18.4%)

M-SLEDAI = Modified SLE Disease Activity Index, BILAG-2004 = British Isles Lupus Assessment Group 2004 (BILAG-2004), SDI = Systemic Lupus International Collaborating Clinics/ American College of Rheumatology damage index, MYR = Malaysian Ringgit

### Content, translational and face validation of the SLEQoL and LupusQoL in Malay

After review of the content and translated version of SLEQoL and LupusQoL, no changes were made with the translated version (M-SLEQoL and M-LupusQoL) provided by the original authors (S1 Appendix-SLEQoL Malay version and S2 Appendix-LupusQoL Malay version).

During the cognitive debriefing of the questionnaires, all subjects concluded that the M- SLEQoL and M-LupusQoL were understandable and no changes were made. The subjects took about 5–10 minutes to complete the questionnaire. The subjects also completed the English version and no difference was found between the Malay and original version of the questionnaires.

Analysis of the 125 SLE patients who completed the M-SLEQoL and M-LupusQoL revealed that the data were not distributed normally by Kolgomorov-Smirnov test with p < 0.05. Table 3 shows the 8 domains of Lupus QoL and 6 domains of SLE QoL and their median values.

**Table 3:** The domains of M-LupusQoL and M-SLEQoL and their median values.

LupusQoL domains	Median (IQR, min-max)	SLEQoL Domains	Median (IQR, min-max)
Physical function	75 (IQR 50, 0–100)	Physical function	9 (IQR 8.5, 6–39)
Pain	75 (IQR 58.3, 0–100)	Symptoms	16 (IQR 12.5, 8–55)
Image	70 (IQR 50, 0–100)	Self-image	21 (IQR 18, 9–62)
Intimate relationship	75 (IQR 100, 0–100)	Treatment	7 (IQR 18, 4–20)
Planning	83.3 (IQR 70.8,0–100)	Activity	19 (IQR 19, 9–59)
Emotional	70.8 (IQR 64.5.0–100)	Mood	9 (IQR 10.5, 4–28)
Fatigue	62.5 (IQR 50, 0–100)		
Burden	66.7 (IQR 66.7, 0–100)		

### Internal consistency

The Cronbach’s α value for M-SLEQOL Malay was 0.890 for all 6 domains which indicates good internal consistency. The Cronbach’s α for domain physical function (PFSQ) was 0.938, activities (ACTVSQ) 0.912, symptoms (SYMPSQ) 0.897, treatment (TRSQ) 0.750 and mood (MOODSQ) 0.926 and self-imaged IMAGESQ (0.907).

The Cronbach’s α value for all 8 domains in M-LupusQOL was 0.944, which indicates good internal consistency. Cronbach’s α for physical function (PFLQ) was 0.957, pain (PAINLQ) 0.916, planning (PLANLQ) 0.968, intimate relationship (INTRELLQ) 0.993, burden to others (BURDENLQ) 0.942, emotional health (EMOLQ) 0.973, body image (IMAGELQ) 0.909 and fatigue (FATIGUELQ) 0.884.

S1 and S2 Tables illustrates the Cronbach’s alpha for the total domain and its sub-items of M-SLEQoL and M-LupusQoL respectively.

### Construct validity using Exploratory Factor Analysis (EFA)

The Kaiser-Mayer-Olkin (KMO) and Bartlett’s test of sphericity (p) for M-SLEQoL was 0.894, p < .001 while M-LupusQoL was 0.946, p<0.001, which indicates sampling was adequate and suitable for EFA. The factor loadings of all the items in M-SLEQoL and M-LupusQoL were acceptable (>0.4).

For M-SLEQoL, the results from the EFA showed 6 components with the first 8 factors Eigen values were greater than 1.0 and the value of the total variance explained by these eight factors was 70.487% that determine number of factors to become domains. Using factor analysis, M-SLEQoL 6 domains physical function (PFSQ), activity (ACTVSQ), symptom (SYMPSQ), treatment (TRSQ), mood (MOODSQ), self image (IMAGESQ) were tested.

6 factors were extracted which represent 6 domains in M-SLEQoL with good factor loading for each item. S3 Table illustrates the Eigen values for M-SLEQoL.

For M-LupusQoL, results from the EFA showed 8 components with the first 4 factors Eigen values greater than 1.0 while the value of the total variance explained by these eight factors was 87.705% that determine the number of factors to become domains. Using factor analysis, M-LupusQOL comprised of 8 domains physical function (PF), pain (PAINLQ), planning (PLANLQ), intimate relationship (INTRELLQ), burden (BURDENLQ), emotion(EMOLQ), image (IMAGELQ) and fatigue (FATIGUELQ) were tested. 8 factors were extracted which represent 8 domains in M-LupusQOL with good factor loading for each item. S4 Table illustrates the Eigen values for M-LupusQoL.

In the rotated component matrix for M-SLEQoL questionnaire, items for domain physical function (PF1 to PF6) were loaded into the same group. Mood domain (MOOD1 until MOOD4) and self-image domain (IMAGE1 until IMAGE8) were loaded into the same group, except item IMAGE9. Items for domain activity (ACTV1 to ACTV 8) were loaded in the same component, except for item ACTV9. Items for treatment (TREATMENT1 to TREATMENT4) were grouped in the same component while items for domain symptom SYMP1 and SYMP4-8 were found in the same component, except for SYMP2 and SYMP3. Component 6 comprised of 2 items ie TR1 and IMAGE9. S5 Table illustrates the factor analysis of 40 items in M-SLEQoL.

In the rotated component matrix of M-LupusQOL, all items in the physical function domain (PF1-PF8), pain (PAIN1-3) and planning (PLAN1-3) were loaded into the same component. All items in the emotion domain (EMO1 to EMO6) were loaded in the same component 2, and all items in the burden domain (BURDEN1 to BURDEN3) were in the same component 3. All items in image domain were loaded in the same component number 4 except for item IMAGE4. All items in fatigue domain were grouped into the same component except for item FATIGUE3. Inter-relationship items were loaded in the same component 6. S6 Table illustrates the factor analysis of 34 items in M-LupusQoL.

### Concurrent validity of M-LupusQoL and M-SLEQoL with M-SF36

There were moderate positive correlations between the M-LupusQoL domains physicial function, fatigue and pain with the comparative domains of M-SF36 (r_s_ ranging from 0.3 to 0.37). A stronger correlation was found in the emotion domain, as illustrated in Table 4.

**Table 4:** Correlation between the comparable domain in M-SLEQoL and M-LupusQoL with M-SF36.

SLEQoL domain	Median (IQR)	M-SF36 domain	Median (IQR, min-max)	r_s_ *(p value)*
Physical function	9 (IQR 8.5	Physical function	70 (IQR 43, 0–100)	-0.538, <0.001
Self-image	21 (IQR 18)	Social functioning	75 (IQR 50, 0–100)	-0.610, <0.001
Mood	9 (IQR 10.5	Emotional health	70 (IQR 43, 0–100)	-0.563, <0.001
Symptoms	16 (IQR 12.5	Bodily pain	68 (IQR 44, 0–100)	-0.600, <0.001
LupusQoL domain	Median (IQR)	SF 36 domain	Median (IQR, min-max)	r_s_ *(p value)*
Physical function	75 (IQR 50)	Physical function	70 (IQR 43, 0–100)	0.307, <0.001
Fatigue	62.5 (IQR 50	Vitality	55 (IQR 23,1–100)	0.366, <0.001
Emotion	70.8 (IQR 64.5	Emotional health	70 (IQR 43, 0–100)	0.493, <0.001
Pain	75 (IQR 58.3	Bodily pain	68 (IQR 44, 0–100	0.377,<0.001

r_s_ = Correlation coefficient

There were strong negative correlations (r_s_ ranging from −0.50 to −0.61) between the four equal domains in M-SLEQoL and M-SF36, as illustrated in Table 4.

### Discriminant validity and correlation with disease activity and damage

There were weak correlations between physical function, activity, treatment, image, mood domain scores of M-SLEQoL with BILAG-2004 and M-SLEDAI score (r_s_ < 0.3). There was no correlation with damage or SDI score, as illustrated in Table 5. There was no correlation between M-LupusQoL with disease activity and damage index.

**Table 5:** Correlation between M-SLEQoL and M-LupusQoL with disease activity and damage.

Domain	r_s_/ p value	BILAG-2004	M-SLEDAI	SDI score
M-SLEQoL				
Physical function	r_s_	0.24	0.24	0.10
	*p*	0.007*	0.007*	0.28
Activity	r_s_	0.29	0.29	0.05
	*p*	0.001*	0.001*	0.61
Symptom	r_s_	0.15	0.13	-0.06
	*p*	0.11	0.14	0.87
Treatment	r_s_	0.18	0.17	0.02
	*p*	0.04*	0.06	0.87
Self-Image	r_s_	0.18	0.19	0.02
	*p*	0.04*	0.03*	0.83
Mood	r_s_	0.22	0.23	0.02
	*p*	0.02*	0.01*	0.84
M-LupusQoL				
Physical function	r_s_	-0.09	-0.02	-0.06
	*p*	0.35	0.23	0.52
Pain	r_s_	-0.06	-0.06	-0.08
	*p*	0.54	0.48	0.39
Planning	r_s_	-0.07	-0.07	-0.10
	*p*	0.46	0.43	0.27
Inter-relationship	r_s_	0.07	0.08	-0.07
	*p*	0.44	0.36	0.46
Burden	r_s_	-0.16	-0.16	-0.09
	*p*	0.08	0.09	0.31
Emotion	r_s_	-0.09	-0.10	-0.06
	*p*	0.30	0.25	0.53
Image	r_s_	-0.03	-0.05	0.001
	*p*	0.72	0.58	0.99
Fatigue	r_s_	0.02	0.006	-0.02
	*p*	0.85	0.95	0.79

*significant p value at <0.05.

r_s_ = Correlation coefficient

BILAG-2004 = British Isles Lupus Assessment Group index 2004, M-SLEDAI = Modified-SLE Disease Activity index, SDI = SLICC damage index

### The associated factors of M-SLEQoL

In univariate analyses, there were inverse correlations between age and treatment, image, mood domains and total M-SLEQoL, suggesting that younger patients had worse QoL. Disease duration was negatively correlated with physical function, activity, image, and mood domains, which indicate lower QoL in these domains among patients with longer disease duration. Patients with hypertension and musculoskeletal involvement had worse physical function domain (p value 0.05 and 0.07, respectively). In contrast, Muslim patients had better QoL in physical function domain (p<0.05). Musculoskeletal (MSK) involvement was associated with worse QoL in activity domain while Malay and Muslim patients had better QoL in this domain (all P<0.05). Worse symptoms domain was associated with both mucocutaneous and MSK involvement while worse QoL in the image domain was associated with lower income category, being married and haematological involvement (all p<0.05).

In the multivariate linear regression analysis which included all variables with p value of <0.10 in the univariate analysis revealed that MSK involvement was independently associated with lower QoL in physical function, activity and symptom domains. Meanwhile, BILAG-2004 scores were correlated with worse QoL in physical and image domains. Table 6 illustrates the multivariate analysis of the independent factors associated with the different domains in M-SLEQoL.

**Table 6:** Multivariate linear regression analysis of the factors associated with different domains in M-SLEQoL.

Domain	Unstandardized Coefficients		Standardized Coefficients	t	95.0% Confidence Interval for B		*P value*
	Β	Std error	β		Lower bound	Upper bound	
**Physical function**							
BILAG-2004	0.48	0.21	0.49	2.33	0.07	0.89	0.02*
M-SLEDAI	-0.42	0.49	-0.18	-0.85	-1.41	0.56	0.39
Disease duration	-0.09	0.10	-0.08	-0.97	-0.29	0.10	0.34
Muslim	-2.76	1.92	-0.12	-1.44	-6.56	1.05	0.15
Hypertension	-2.70	1.65	-0.14	-1.64	-5.97	0.57	0.10
Musculoskeletal	3.22	1.46	0.19	2.21	0.34	6.09	0.03*
**Activity**							
BILAG-2004	0.58	0.32	0.36	1.79	-0.06	1.22	0.08
M-SLEDAI	-0.07	0.78	-0.02	-0.09	-1.61	1.47	0.93
Age	-0.05	0.10	-0.05	-0.52	-0.24	0.14	0.61
Duration	-0.19	0.17	-0.10	-1.13	-0.52	0.14	0.26
Musculoskeletal	7.14	2.32	0.26	3.08	2.60	11.73	0.003*
Muslim	-10.36	8.59	-0.29	-1.21	-27.37	6.66	0.23
Malay	4.014	8.228	0.12	0.49	-12.28	20.31	0.63
**Symptom**							
Mucocutaneous	2.17	1.68	0.11	1.29	-1.15	5.49	0.20
Musculoskeletal	4.27	1.86	0.20	2.30	0.59	7.94	0.02*
Low income	4.93	2.12	0.21	2.33	0.74	9.13	0.02*
**Image**							
BILAG-2004	0.72	0.32	0.46	2.25	0.09	1.35	0.03*
M-SLEDAI	-0.85	0.78	-0.23	-1.09	-2.39	0.69	0.28
Age	-0.18	0.11	-0.17	-1.69	-0.39	0.03	0.09
Duration	-0.27	0.17	-0.14	-1.60	-0.60	0.06	0.11
Low income	3.99	2.96	0.13	1.35	-1.88	9.85	0.18
Higher education	-2.54	2.38	-0.10	-1.07	-7.25	2.17	0.29
Muslim	-6.24	2.91	-0.18	-2.15	-12.00	-0.49	0.03*
Married	-1.08	2.42	-0.04	-0.45	-5.88	3.71	0.66
Renal	1.01	2.36	0.04	0.43	-3.66	5.67	0.67
Hematological	2.30	2.19	0.09	1.05	-2.05	6.65	0.29
**Treatment**							
BILAG-2004	0.15	0.12	0.28	1.26	-0.08	0.38	0.21
M-SLEDAI	-0.10	0.28	-0.08	-0.35	-0.66	0.46	0.73
Age	-0.06	0.04	-0.16	-1.66	-0.13	0.01	0.10
Duration	-0.02	0.06	-0.03	-0.31	-0.14	0.10	0.76
Higher education	-0.96	0.79	-0.11	-1.23	-2.52	0.59	0.23
**Mood**							
BILAG-2004	0.20	0.17	0.26	1.21	-0.13	0.54	0.20
M-SLEDAI	-0.10	0.41	-0.05	-0.25	-0.91	0.70	0.80
Age	-0.11	0.05	-0.20	-2.24	-0.21	-0.01	0.03*
Duration	-0.10	0.09	-0.11	-1.16	-0.27	0.072	0.25
Low socioeconomic	2.07	1.35	0.13	1.53	-0.61	4.75	0.13
Haematological	0.89	1.13	0.07	0.79	-1.35	3.12	0.43

*significant p value at <0.05

### The associated factors of M-LupusQoL

Patients with musculoskeletal (MSK) involvement had worse pain and planning QoL scores [66.67(IQR66.67) vs 83.3(IQR45.83), p = 0.017] and [75(IQR75) vs 100(IQR41.67), p = 0.01], respectively. Musculoskeletal involvement remained as a significant factor in the multivariate linear regression analysis which included other variables that were significant at p<0.10 in the initial univariate analysis.

Patients with MSK, muco-cutaneous, and neuropsychiatric lupus (NPSLE) tend to have lower scores in the fatigue domain, with p<0.1. In the multivariate linear regression analysis, MSK and NPSLE remained to be significantly correlated with low fatigue domain scores.

Patients with renal involvement had better intimate relationship QoL score in the univariate analysis [100 (IQR 62.5) vs 31.25(IQR100), p = 0.002]. However, it was no longer significant in the multivariate linear regression analysis. No associations or correlations were found with other socio-demographic, disease characteristics and patients’ co-morbidities.

## Discussion

The assessment of QoL has become an essential component apart from the disease remission and prevention of organ damage in SLE. Therefore, patient reported outcome (PRO) instruments are designed to assess their QoL. QoL affects several aspects of the patients’ lives including their work [31], daily-living activities [28] and their coping mechanisms [32]. By exploring the multi-dimensional aspects of the patients, this will facilitate better communication with the multidisciplinary team to optimize their health status [33].

Lupus specific measures are superior to generic scales in the assessing the QoL [34].

Our study was the first in Malaysia to validate lupus-specific QoL instruments and to assess the impact of demographic and disease-related characteristics on HRQoL using the validated translated Malay version of SLEQoL and LupusQoL (M-SLEQoL and M-LupusQoL). In our study, both M-SLEQoL and M-LupusQoL were demonstrated to have a good internal consistency reliability.

Both instruments also have concurrent validity with the comparable individual domains in the generic QoL SF-36. M-SLEQoL demonstrates stronger correlations with SF36 and this finding is in contrast with the initial development and validation of SLEQoL, which showed poor concurrent validity with the SF-36 [12]. On the other hand, the initial development and validation of LupusQoL questionnaire has demonstrated good concurrent validity with SF-36, with very strong correlation coefficient (r_s_ = 0.71 to 0.79) [11]. Similar results were obtained in the U.S. [35] and Spanish [36] validation studies.

There are inconsistencies in the relationship between QoL in SLE patients with disease activity [16]. The initial development and validation of SLEQoL in Singapore found no correlation with disease activity [12]. Other cross-cultural adaptation studies in Brazil also found poor correlation between SLEQoL and disease activity [37]. However, in our study, SLEQoL was found to be correlated with disease activity and the BILAG scores were the independent predictors of the physical function and image domains of SLEQoL. Cross-cultural validation study in Thailand also found significant correlation between disease activity measured with SLEDAI-2K with total SLEQoL-TH score, activity and symptom domains. Meanwhile, the Arabic SLEQoL demonstrates correlation between SLEDAI-2K with the total score, treatment and self-image domain of SLEQoL.

In contrast, poor correlation was found between LupusQoL and disease activity among our cohort of patients. However, the initial development [11]and other linguistic validation studies of the LupusQoL [38–40] have demonstrated a significant correlation with disease activity. This discrepancy can be explained by the difference in the recall period between these 2 instruments. It should also be noted that our study sample size was small and thus lack of heterogeneity as not many of our patients had active disease or considerable disease damage. Therefore, further larger studies are warranted to further include a wide spectrum of disease activity and damage of the SLE.

In our study, both SLEQoL and LupusQoL had no correlation with disease damage. This is consistent with the validation study in Brazil [37]. In contrast, SLEQoL cross-cultural validation study in Thailand [14] showed significant correlation between SLICC/ACR and the physical function domain while the Arabic version of SLEQoL showed significant correlation with physical, symptoms, treatment and self-image domains [15]. In contrast to our study, LupusQoL in other studies in the UK [11], US [35], China [39], Mexico [41], Iran [42] and Spanish [43] the were found to be able to discriminate based on damage (SDI scores damage). However, majority of these studies demonstrates weak correlations with the damage index.

Our study found that MSK involvement was the independent predictor of physical function, activity and symptom domains in M-SLEQoL. Meanwhile, MSK and NPSLE were the independent predictor of low QoL in fatigue domain of M-LupusQoL. Both MSK and NPSLE were indeed associated with lower QoL in various other studies [44–46].

## Strength and limitations

Our study was the first to perform the validation and cross-cultural adaption of the two commonly used SLE-specific health related questionnaires in Malay language. We have shown that both M-SLEQoL and M-LupusQoL had good internal consistencies, acceptable factor analysis and concurrent validity with the M-SF36. However, there are several limitations in our study. Our study was a cross-sectional study so we did not perform test-retest reliability. During the cognitive debriefing, we only obtained subjective feedback from the respondents and did not measure the Item-face validity index (I-FVI) and (Scale-face validity index) S-FVI. The sample size in our study also was small so we did not perform confirmatory factor analysis (CFA). Instead we use the exploratory factor analysis (EFA) for preliminary determination of the questionnaires’ construct validity [47]. Therefore, a larger study is needed in the future to further confirm the validity in a new data set of patients. Our study also could not establish any responsiveness of the M-SLEQoL and M-LupusQoL to changes. In addition, many other possible confounding variables that might influence QoL of the patients were not measured in our study. For instance co-morbidity fibromyalgia has been shown to be an independent and important HRQoL predictor [16, 41] were not measured in this study. Different types of treatment and their side-effects were also not studied.

## Conclusion

The results of this study provide evidence regarding the reliability and validity of the Malays version of SLEQoL and LupusQoL questionnaires in our SLE population. However, M-LupusQoL has better discriminant validity as it functions independently from measures of disease activity and damage. The QoL of patients with musculoskeletal (MSK) involvement were mostly affected as it was associated with lower M-SLEQoL in physical function, activity and symptom domains, and fatigue domain in M-LupusQoL.

## Supporting information

S1 File Item-content validity index (I-CVI) and scale content validity index of M-SLEQoL and M-LupusQoL. (XLSX)

S2 File Raw data. (XLSX)

S1 Table Cronbach’s alpha for the total domain and its sub-items of SLEQoL. (DOCX)

S2 Table Cronbach’s alpha for the total scale and its subscales’ and item analysis of LupusQoL. (DOCX)

S3 Table Eigen values for M-SLEQoL. (DOCX)

S4 Table Eigen values for M-LupusQoL. (DOCX)

S5 Table Exploratory Factor Analysis of 40 items in M-SLEQoL (values below 0.4 are suppressed). (DOCX)

S6 Table Exploratory factor analysis of the 34 items in M-LupusQoL (values below 0.4 are suppressed). (DOCX)

S1 Appendix SLEQoL Malay version. (DOC)

S2 Appendix LupusQoL Malay version. (DOCX)

## References

[1] Carter EE, Barr SG, Clarke AE. The global burden of SLE: prevalence, health disparities and socioeconomic impact. Nature Reviews Rheumatology. 2016;12(10):605–20. doi: 10.1038/nrrheum.2016.137 27558659

[2] Hyphantis T, Palieraki K, Voulgari PV, Tsifetaki N, Drosos AA. Coping with health-stressors and defence styles associated with health-related quality of life in patients with systemic lupus erythematosus. Lupus. 2011;20(9):893–903. Epub 2011/05/13. doi: 10.1177/0961203311398264 . 21562019

[3] McElhone K, Abbott J, Teh LS. A review of health related quality of life in systemic lupus erythematosus. Lupus. 2006;15(10):633–43. Epub 2006/11/24. doi: 10.1177/0961203306071710 . 17120589

[4] Strand V, Gladman D, Isenberg D, Petri M, Smolen J, Tugwell P. Endpoints: consensus recommendations from OMERACT IV. Outcome Measures in Rheumatology. Lupus. 2000;9(5):322–7. doi: 10.1191/096120300678828424 . 10878722

[5] Guidance for industry: patient-reported outcome measures: use in medical product development to support labeling claims: draft guidance. Health Qual Life Outcomes. 2006;4:79. Epub 20061011. doi: 10.1186/1477-7525-4-79 . 17034633 PMC1629006

[6] European Medicines Agency: Guideline on clinical investigation of medicinal products for the treatment of systemic lupus erythematosus and lupus nephritis. In: (CHMP) CfMPfHu, editor. 2015.

[7] Izadi Z, Gandrup J, Katz PP, Yazdany J. Patient-reported outcome measures for use in clinical trials of SLE: a review. Lupus Science & Medicine. 2018;5(1):e000279. doi: 10.1136/lupus-2018-000279 30167315 PMC6109821

[8] Sararaks S, Azman AB, Low LL, Rugayah B, Aziah AM, Hooi LN, et al. Validity and reliability of the SF-36: the Malaysian context. Med J Malaysia. 2005;60(2):163–79. Epub 2005/08/24. . 16114157

[9] Yazdany J. Health-related quality of life measurement in adult systemic lupus erythematosus: Lupus Quality of Life (LupusQoL), Systemic Lupus Erythematosus-Specific Quality of Life Questionnaire (SLEQOL), and Systemic Lupus Erythematosus Quality of Life Questionnaire (L-QoL). Arthritis Care Res (Hoboken). 2011;63 Suppl 11(0 11):S413–S9. doi: 10.1002/acr.20636 . 22588761 PMC3437754

[10] Toloza SM, Jolly M, Alarcón GS. Quality-of-life measurements in multiethnic patients with systemic lupus erythematosus: cross-cultural issues. Curr Rheumatol Rep. 2010;12(4):237–49. doi: 10.1007/s11926-010-0110-5 . 20461481

[11] McElhone K, Abbott J, Shelmerdine J, Bruce IN, Ahmad Y, Gordon C, et al. Development and validation of a disease-specific health-related quality of life measure, the LupusQol, for adults with systemic lupus erythematosus. Arthritis Rheum. 2007;57(6):972–9. doi: 10.1002/art.22881 . 17665467

[12] Leong KP, Kong KO, Thong BY, Koh ET, Lian TY, Teh CL, et al. Development and preliminary validation of a systemic lupus erythematosus-specific quality-of-life instrument (SLEQOL). Rheumatology (Oxford). 2005;44(10):1267–76. Epub 20050329. doi: 10.1093/rheumatology/keh605 . 15797980

[13] Kong KO, Ho HJ, Howe HS, Thong BY, Lian TY, Chng HH, et al. Cross-cultural adaptation of the Systemic Lupus Erythematosus Quality of Life Questionnaire into Chinese. Arthritis Rheum. 2007;57(6):980–5. doi: 10.1002/art.22911 . 17665463

[14] Kasitanon N, Wangkaew S, Puntana S, Sukitawut W, Leong KP, Louthrenoo W. The reliability, validity and responsiveness of the Thai version of Systemic Lupus Erythematosus Quality of Life (SLEQOL-TH) instrument. Lupus. 2013;22(3):289–96. Epub 20130128. doi: 10.1177/0961203312474702 . 23358871

[15] Aziz MM, Galal MAA, Elzohri MH, El-Nouby F, Leong KP. Cross-cultural adaptation and validation of Systemic Lupus Erythematosus Quality of Life questionnaire into Arabic. Lupus. 2018;27(5):780–7. doi: 10.1177/0961203317747714 . 29308728

[16] Elera-Fitzcarrald C, Fuentes A, González LA, Burgos PI, Alarcón GS, Ugarte-Gil MF. Factors affecting quality of life in patients with systemic lupus erythematosus: important considerations and potential interventions. Expert Rev Clin Immunol. 2018;14(11):915–31. Epub 20181008. doi: 10.1080/1744666X.2018.1529566 . 30266076

[17] Hochberg MC. Updating the American College of Rheumatology revised criteria for the classification of systemic lupus erythematosus. Arthritis Rheum. 1997;40(9):1725. doi: 10.1002/art.1780400928 . 9324032

[18] Petri M, Orbai AM, Alarcón GS, Gordon C, Merrill JT, Fortin PR, et al. Derivation and validation of the Systemic Lupus International Collaborating Clinics classification criteria for systemic lupus erythematosus. Arthritis Rheum. 2012;64(8):2677–86. doi: 10.1002/art.34473 . 22553077 PMC3409311

[19] Costello AB, Osborne J. Best Practices in Exploratory Factor Analysis: Four Recommendations for Getting the Most From Your Analysis. Practical Assessment, Research & Evaluation. 2005;10:1–9.

[20] Isenberg DA, Rahman A, Allen E, Farewell V, Akil M, Bruce IN, et al. BILAG 2004. Development and initial validation of an updated version of the British Isles Lupus Assessment Group’s disease activity index for patients with systemic lupus erythematosus. Rheumatology (Oxford). 2005;44(7):902–6. doi: 10.1093/rheumatology/keh624 . 15814577

[21] Gyori N, Giannakou I, Chatzidionysiou K, Magder L, van Vollenhoven RF, Petri M. Disease activity patterns over time in patients with SLE: analysis of the Hopkins Lupus Cohort. Lupus Sci Med. 2017;4(1):e000192. Epub 2017/03/01. doi: 10.1136/lupus-2016-000192 . 28243457 PMC5307372

[22] Yee CS, Cresswell L, Farewell V, Rahman A, Teh LS, Griffiths B, et al. Numerical scoring for the BILAG-2004 index. Rheumatology (Oxford). 2010;49(9):1665–9. doi: 10.1093/rheumatology/keq026 . 20181671 PMC2919194

[23] Gladman D, Ginzler E, Goldsmith C, Fortin P, Liang M, Urowitz M, et al. The development and initial validation of the Systemic Lupus International Collaborating Clinics/American College of Rheumatology damage index for systemic lupus erythematosus. Arthritis Rheum. 1996;39(3):363–9. Epub 1996/03/01. doi: 10.1002/art.1780390303 . 8607884

[24] Revicki D. Internal Consistency Reliability. In: Michalos AC, editor. Encyclopedia of Quality of Life and Well-Being Research. Dordrecht: Springer Netherlands; 2014. p. 3305–6.

[25] Mohamad Adam B. H Y, & Lee KY. A Step By Step Guide To Questionnaire Validation Research: Kuala Lumpur, Malaysia: Institute for Clinical Research, NIH MY; 2022.

[26] Musa AF, Yasin MSM, Smith J, Yakub MA, Nordin RB. The Malay version of SF-36 health survey instrument: testing data quality, scaling assumptions, reliability and validity in post-coronary artery bypass grafting (CABG) surgery patients at the National Heart Institute (Institut Jantung Negara—IJN), Kuala Lumpur. Health and Quality of Life Outcomes. 2021;19(1):50. doi: 10.1186/s12955-020-01658-9 33563262 PMC7874445

[27] Abu Bakar F, Sazliyana Shaharir S, Mohd R, Mohamed Said MS, Rajalingham S, Wei Yen K. Burden of Systemic Lupus Erythematosus on Work Productivity and Daily Living Activity: A Cross-Sectional Study Among Malaysian Multi-Ethnic Cohort. Arch Rheumatol. 2020;35(2):205–13. Epub 2020/08/28. doi: 10.46497/ArchRheumatol.2020.7405 . 32851369 PMC7406156

[28] Abu Bakar F, Shaharir SS, Mohd R, Kamaruzaman L, Mohamed Said MS. Work disability in a multi-ethnic Malaysian systemic lupus erythematosus cohort: A cross-sectional study. Int J Rheum Dis. 2019;22(6):1002–7. Epub 2019/04/11. doi: 10.1111/1756-185X.13572 . 30968556

[29] Saddki N, Mohamad H, Yusof N, Mohamad D, Mokhtar N, Wan bakar Wz. Validity and reliability of the Malay version of Sleep Apnea Quality of Life Index—preliminary results. Health and quality of life outcomes. 2013;11:100. doi: 10.1186/1477-7525-11-100 23786866 PMC3710083

[30] Guadagnoli E, Velicer WF. Relation of sample size to the stability of component patterns. Psychological Bulletin. 1988;103:265–75. doi: 10.1037/0033-2909.103.2.265 3363047

[31] Abu Bakar F, Shaharir SS, Mohd R, Kamaruzaman L, Mohamed Said MS. Work disability in a multi-ethnic Malaysian systemic lupus erythematosus cohort: A cross-sectional study. International Journal of Rheumatic Diseases. 2019;22(6):1002–7. doi: 10.1111/1756-185X.13572 30968556

[32] Fischin J, Chehab G, Richter JG, Fischer-Betz R, Winkler-Rohlfing B, Willers R, et al. Factors associated with pain coping and catastrophising in patients with systemic lupus erythematosus: a cross-sectional study of the LuLa-cohort. Lupus Sci Med. 2015;2(1):e000113. Epub 20151112. doi: 10.1136/lupus-2015-000113 . 26629351 PMC4654099

[33] Mahieu M, Yount S, Ramsey-Goldman R. Patient-Reported Outcomes in Systemic Lupus Erythematosus. Rheum Dis Clin North Am. 2016;42(2):253–63. Epub 2016/03/17. doi: 10.1016/j.rdc.2016.01.001 . 27133488 PMC4853651

[34] Yee CS, McElhone K, Teh LS, Gordon C. Assessment of disease activity and quality of life in systemic lupus erythematosus—New aspects. Best Pract Res Clin Rheumatol. 2009;23(4):457–67. doi: 10.1016/j.berh.2009.01.007 . 19591777

[35] Jolly M, Pickard AS, Wilke C, Mikolaitis RA, Teh LS, McElhone K, et al. Lupus-specific health outcome measure for US patients: the LupusQoL-US version. Ann Rheum Dis. 2010;69(1):29–33. doi: 10.1136/ard.2008.094763 . 19126561

[36] González-Rodríguez V, Peralta-Ramírez MI, Navarrete-Navarrete N, Callejas-Rubio JL, Santos Ruiz AM, Khamashta M. [Adaptation and validation of the Spanish version of a disease-specific quality of life measure in patients with systemic lupus erythematosus: the Lupus quality of life]. Med Clin (Barc). 2010;134(1):13–6. Epub 20090731. doi: 10.1016/j.medcli.2009.04.045 . 19647276

[37] Freire EA, Bruscato A, Leite DR, Sousa TT, Ciconelli RM. Translation into Brazilian Portuguese, cultural adaptation and validatation of the systemic lupus erythematosus quality of life questionnaire (SLEQOL). Acta Reumatol Port. 2010;35(3):334–9. . 20975636

[38] Conti F, Perricone C, Reboldi G, Gawlicki M, Bartosiewicz I, Pacucci V, et al. Validation of a disease-specific health-related quality of life measure in adult Italian patients with systemic lupus erythematosus: LupusQoL-IT. Lupus. 2014;23(8):743–51. doi: 10.1177/0961203314524466 . 24569393

[39] Wang S-l, Wu B, Leng L, Bucala R, Lu L-j. Validity of LupusQoL-China for the Assessment of Health Related Quality of Life in Chinese Patients with Systemic Lupus Erythematosus. PLoS One. 2013;8(5):e63795. doi: 10.1371/journal.pone.0063795 23717486 PMC3662722

[40] Devilliers H, Amoura Z, Besancenot JF, Bonnotte B, Pasquali JL, Wahl D, et al. LupusQoL-FR is valid to assess quality of life in patients with systemic lupus erythematosus. Rheumatology (Oxford). 2012;51(10):1906–15. Epub 20120722. doi: 10.1093/rheumatology/kes165 . 22826305

[41] Etchegaray-Morales I, Méndez-Martínez S, Jiménez-Hernández C, Mendoza-Pinto C, Alonso-García NE, Montiel-Jarquín A, et al. Factors Associated with Health-Related Quality of Life in Mexican Lupus Patients Using the LupusQol. PLoS One. 2017;12(1):e0170209. Epub 20170123. doi: 10.1371/journal.pone.0170209 . 28114336 PMC5256863

[42] Hosseini N, Bonakdar ZS, Gholamrezaei A, Mirbagher L. Linguistic Validation of the LupusQoL for the Assessment of Quality of Life in Iranian Patients with Systemic Lupus Erythematosus. Int J Rheumatol. 2014;2014:151530. doi: 10.1155/2014/151530 . 25313310 PMC4172886

[43] Carrión-Nessi FS, Marcano-Rojas MV, Romero Arocha SR, Mendoza Millán DL, Forero-Peña DA, Antuarez-Magallanes AW, et al. Impact of demographic, clinical, and treatment compliance characteristics on quality of life of Venezuelan patients with systemic lupus erythematosus. BMC Rheumatology. 2022;6(1):2. doi: 10.1186/s41927-021-00232-0 34983688 PMC8725562

[44] Louthrenoo W, Kasitanon N, Morand E, Kandane-Rathnayake R. Comparison of performance of specific (SLEQOL) and generic (SF36) health-related quality of life questionnaires and their associations with disease status of systemic lupus erythematosus: a longitudinal study. Arthritis Res Ther. 2020;22(1):8. Epub 20200110. doi: 10.1186/s13075-020-2095-4 . 31924267 PMC6954627

[45] Piga M, Congia M, Gabba A, Figus F, Floris A, Mathieu A, et al. Musculoskeletal manifestations as determinants of quality of life impairment in patients with systemic lupus erythematosus. Lupus. 2018;27(2):190–8. Epub 20170615. doi: 10.1177/0961203317716319 . 28618891

[46] Natalucci F, Ceccarelli F, Cipriano E, Perricone C, Olivieri G, Pirone C, et al. Joint involvement influences quality of life in systemic lupus erythematosus patients. Lupus. 2021;30(3):478–83. Epub 20201215. doi: 10.1177/0961203320979039 . 33323011

[47] Orcan F. Exploratory and Confirmatory Factor Analysis: Which One to Use First? Eğitimde ve Psikolojide Ölçme ve Değerlendirme Dergisi. 2018:414–21. doi: 10.21031/epod.394323

