# Supplementary material for: Validation and the associated factors of the Malay version of systemic lupus erythematosus-specific health-related quality of life questionnaires (SLEQoL and LupusQoL)
Source: PLoS One. 2023 May 15;18(5):e0285461. doi: 10.1371/journal.pone.0285461 (PMC10184909; doi:10.1371/journal.pone.0285461)
Supplement: S1 Table — (DOCX) [file pone.0285461.s003.docx]

Table S1. Cronbach’s alpha for the total domain and its sub-items of SLEQoL.

| Domain/ Item | Scale Mean if Item Deleted | Scale Variance if Item Deleted | Corrected Item-Total Correlation | Squared Multiple Correlation | Cronbach's Alpha if Item Deleted | Total  Cronbach's |
| --- | --- | --- | --- | --- | --- | --- |
| Physical function (PF) | | | | | |  |
| PF1 | 10.4480 | 45.056 | .849 | .812 | .923 |  |
| PF2 | 10.1520 | 41.211 | .922 | .901 | .912 |  |
| PF3 | 10.4960 | 44.575 | .849 | .834 | .922 |  |
| PF4 | 10.3280 | 43.125 | .901 | .871 | .916 |  |
| PF5 | 10.5840 | 45.358 | .835 | .785 | .925 |  |
| PF6 | 9.1920 | 43.076 | .628 | .481 | .959 |  |
| Activities (ACTV) | | | | | |  |
| ACTV1 | 19.8800 | 123.800 | .817 | .839 | .894 |  |
| ACTV2 | 19.7760 | 121.111 | .871 | .893 | .890 |  |
| ACTV3 | 20.0960 | 124.394 | .803 | .802 | .895 |  |
| ACTV4 | 20.4880 | 130.994 | .772 | .688 | .899 |  |
| ACTV5 | 19.1680 | 121.318 | .734 | .589 | .900 |  |
| ACTV6 | 20.1200 | 137.445 | .410 | .345 | .922 |  |
| ACTV7 | 19.8000 | 123.339 | .793 | .691 | .895 |  |
| ACTV8 | 19.3600 | 128.345 | .618 | .524 | .908 |  |
| ACTV9 | 19.9360 | 130.351 | .535 | .400 | .914 |  |
| Symptom (SYMP) | | | | | |  |
| SYMP1 | 16.1280 | 72.645 | .712 | .554 | .881 |  |
| SYMP2 | 16.6320 | 75.509 | .658 | .532 | .886 |  |
| SYMP3 | 15.8320 | 69.173 | .729 | .769 | .879 |  |
| SYMP4 | 15.9280 | 68.358 | .805 | .814 | .871 |  |
| SYMP5 | 16.5600 | 73.652 | .652 | .556 | .886 |  |
| SYMP6 | 17.1040 | 79.513 | .523 | .350 | .897 |  |
| SYMP7 | 16.7440 | 73.966 | .617 | .622 | .890 |  |
| SYMP8 | 16.1680 | 67.641 | .740 | .586 | .878 |  |
| Treatment (TR) | | | | | |  |
| TR1 | 6.3200 | 12.316 | .451 | .218 | .741 |  |
| TR2 | 5.9600 | 10.942 | .570 | .348 | .679 |  |
| TR3 | 6.0960 | 10.926 | .597 | .398 | .664 |  |
| TR4 | 5.8160 | 10.345 | .569 | .344 | .680 |  |
| Mood (MOOD) | | | | | |  |
| MOOD1 | 8.6560 | 27.211 | .711 | .509 | .941 |  |
| MOOD2 | 7.7840 | 22.138 | .890 | .808 | .883 |  |
| MOOD3 | 8.0160 | 22.967 | .881 | .796 | .886 |  |
| MOOD4 | 7.7040 | 21.662 | .851 | .744 | .899 |  |
| Self-Image (IMAGE) | | | | | |  |
| IMAGE1 | 21.0880 | 122.065 | .735 | .646 | .893 |  |
| IMAGE2 | 22.0080 | 140.282 | .544 | .450 | .906 |  |
| IMAGE3 | 21.2000 | 122.194 | .799 | .829 | .888 |  |
| IMAGE4 | 21.5760 | 125.956 | .775 | .796 | .891 |  |
| IMAGE5 | 20.3280 | 116.303 | .771 | .675 | .891 |  |
| IMAGE6 | 20.7360 | 121.712 | .767 | .665 | .890 |  |
| IMAGE7 | 19.9680 | 120.757 | .771 | .756 | .890 |  |
| IMAGE8 | 19.6720 | 119.722 | .771 | .822 | .890 |  |
| IMAGE9 | 22.3520 | 153.601 | .143 | .139 | .923 |  |
