# Supplementary material for: Validation and the associated factors of the Malay version of systemic lupus erythematosus-specific health-related quality of life questionnaires (SLEQoL and LupusQoL)
Source: PLoS One. 2023 May 15;18(5):e0285461. doi: 10.1371/journal.pone.0285461 (PMC10184909; doi:10.1371/journal.pone.0285461)
Supplement: S2 Table — (DOCX) [file pone.0285461.s004.docx]

Table S2. Cronbach’s alpha for the total scale and its subscales’ and item analysis of LupusQoL

| Domain/ Item | Scale Mean if Item Deleted | Scale Variance if Item Deleted | Corrected Item-Total Correlation | Squared Multiple Correlation | Cronbach's Alpha if Item Deleted | Total  Cronbach's |
| --- | --- | --- | --- | --- | --- | --- |
| Physical Function (PF) | | | | | | .957 |
| PF1 | 18.1040 | 86.046 | .694 | .557 | .960 |  |
| PF2 | 17.8640 | 80.280 | .915 | .883 | .946 |  |
| PF3 | 17.6080 | 81.724 | .870 | .888 | .949 |  |
| PF4 | 17.6320 | 80.541 | .905 | .888 | .947 |  |
| PF5 | 18.0000 | 82.468 | .827 | .730 | .952 |  |
| PF6 | 17.8720 | 81.209 | .898 | .843 | .947 |  |
| PF7 | 18.0960 | 84.039 | .828 | .788 | .952 |  |
| PF8 | 18.1280 | 85.338 | .759 | .634 | .956 |  |
| Pain (PAIN) | | | | | | .916 |
| PAIN1 | 4.9440 | 7.989 | .807 | .652 | .898 |  |
| PAIN2 | 5.0160 | 7.564 | .846 | .719 | .867 |  |
| PAIN3 | 4.8080 | 7.834 | .840 | .711 | .872 |  |
| Planning (PLAN) | | | | | | .968 |
| PLAN1 | 5.3680 | 8.654 | .931 | .872 | .953 |  |
| PLAN2 | 5.3520 | 8.714 | .943 | .891 | .944 |  |
| PLAN3 | 5.3760 | 8.785 | .918 | .845 | .961 |  |
| Inter-relationship (INTREL) | | | | | | .993 |
| INTREL1 | 2.4630 | 2.718 | .986 | .973 | . |  |
| INTREL2 | 2.4630 | 2.756 | .986 | .973 | . |  |
| Burden (BURDEN) | | | | | | .942 |
| BURDEN1 | 4.5600 | 9.039 | .872 | .795 | .919 |  |
| BURDEN2 | 4.7200 | 8.945 | .919 | .849 | .883 |  |
| BURDEN3 | 4.6080 | 9.160 | .844 | .730 | .941 |  |
| Emotion (EMO) | | | | | | .973 |
| EMO1 | 12.4400 | 46.990 | .869 | .787 | .972 |  |
| EMO2 | 12.3600 | 47.152 | .883 | .818 | .970 |  |
| EMO3 | 12.4720 | 46.171 | .943 | .920 | .964 |  |
| EMO4 | 12.5120 | 46.800 | .936 | .935 | .965 |  |
| EMO5 | 12.5760 | 46.149 | .947 | .951 | .964 |  |
| EMO6 | 12.5200 | 46.913 | .886 | .805 | .970 |  |
| Image (IMAGE) | | | | | | .909 |
| IMAGE1 | 10.1520 | 25.501 | .838 | .739 | .874 |  |
| IMAGE2 | 10.0480 | 26.240 | .805 | .716 | .881 |  |
| IMAGE3 | 10.1600 | 25.329 | .805 | .662 | .881 |  |
| IMAGE4 | 10.1200 | 27.171 | .701 | .548 | .903 |  |
| IMAGE5 | 10.1440 | 27.366 | .703 | .509 | .902 |  |
| Fatigue (FATIGUE) | | | | | | .884 |
| FATIGUE1 | 7.0880 | 13.597 | .815 | .696 | .825 |  |
| FATIGUE2 | 7.2080 | 14.214 | .760 | .619 | .846 |  |
| FATIGUE3 | 7.0400 | 14.119 | .656 | .432 | .889 |  |
| FATIGUE4 | 7.0800 | 13.881 | .769 | .607 | .842 |  |
