# Supplementary material for: Validation and the associated factors of the Malay version of systemic lupus erythematosus-specific health-related quality of life questionnaires (SLEQoL and LupusQoL)
Source: PLoS One. 2023 May 15;18(5):e0285461. doi: 10.1371/journal.pone.0285461 (PMC10184909; doi:10.1371/journal.pone.0285461)
Supplement: S3 Table — (DOCX) [file pone.0285461.s005.docx]

Table S3. Eigen values for M-SLEQoL

| Table 9h Total Variance Explained | | | | | | |
| --- | --- | --- | --- | --- | --- | --- |
| Component | Initial Eigenvalues | | | Rotation Sums of Squared Loadings | | |
|  | Total | % of Variance | Cumulative % | Total | % of Variance | Cumulative % |
| 1 | 18.619 | 46.547 | 46.547 | 7.256 | 18.141 | 18.141 |
| 2 | 3.202 | 8.005 | 54.551 | 5.847 | 14.617 | 32.758 |
| 3 | 2.178 | 5.445 | 59.997 | 5.102 | 12.754 | 45.512 |
| 4 | 1.611 | 4.028 | 64.025 | 4.135 | 10.337 | 55.848 |
| 5 | 1.341 | 3.353 | 67.378 | 4.126 | 10.315 | 66.163 |
| 6 | 1.244 | 3.109 | 70.487 | 1.730 | 4.324 | 70.487 |
| 7 | 1.112 | 2.779 | 73.267 |  |  |  |
| 8 | 1.066 | 2.664 | 75.930 |  |  |  |
| 9 | .883 | 2.209 | 78.139 |  |  |  |
| 10 | .849 | 2.123 | 80.262 |  |  |  |
| 11 | .750 | 1.876 | 82.138 |  |  |  |
| 12 | .714 | 1.785 | 83.923 |  |  |  |
| 13 | .642 | 1.605 | 85.528 |  |  |  |
| 14 | .593 | 1.483 | 87.011 |  |  |  |
| 15 | .541 | 1.353 | 88.364 |  |  |  |
| 16 | .464 | 1.161 | 89.525 |  |  |  |
| 17 | .410 | 1.025 | 90.550 |  |  |  |
| 18 | .382 | .956 | 91.506 |  |  |  |
| 19 | .357 | .892 | 92.397 |  |  |  |
| 20 | .317 | .792 | 93.190 |  |  |  |
| 21 | .313 | .783 | 93.973 |  |  |  |
| 22 | .278 | .696 | 94.668 |  |  |  |
| 23 | .243 | .607 | 95.275 |  |  |  |
| 24 | .227 | .569 | 95.844 |  |  |  |
| 25 | .199 | .498 | 96.342 |  |  |  |
| 26 | .179 | .447 | 96.789 |  |  |  |
| 27 | .177 | .442 | 97.231 |  |  |  |
| 28 | .155 | .388 | 97.618 |  |  |  |
| 29 | .144 | .359 | 97.977 |  |  |  |
| 30 | .128 | .320 | 98.298 |  |  |  |
| 31 | .119 | .298 | 98.596 |  |  |  |
| 32 | .106 | .266 | 98.862 |  |  |  |
| 33 | .093 | .234 | 99.095 |  |  |  |
| 34 | .084 | .209 | 99.304 |  |  |  |
| 35 | .072 | .181 | 99.485 |  |  |  |
| 36 | .063 | .158 | 99.643 |  |  |  |
| 37 | .045 | .113 | 99.756 |  |  |  |
| 38 | .039 | .098 | 99.854 |  |  |  |
| 39 | .032 | .081 | 99.935 |  |  |  |
| 40 | .026 | .065 | 100.000 |  |  |  |
| Extraction Method: Principal Component Analysis. | | | | | | |
