# Supplementary material for: Validation and the associated factors of the Malay version of systemic lupus erythematosus-specific health-related quality of life questionnaires (SLEQoL and LupusQoL)
Source: PLoS One. 2023 May 15;18(5):e0285461. doi: 10.1371/journal.pone.0285461 (PMC10184909; doi:10.1371/journal.pone.0285461)
Supplement: S4 Table — (DOCX) [file pone.0285461.s006.docx]

Table S4. Eigen values for M-LupusQoL

| Table 9c Total Variance Explained | | | | | | |
| --- | --- | --- | --- | --- | --- | --- |
| Component | Initial Eigenvalues | | | Rotation Sums of Squared Loadings | | |
|  | Total | % of Variance | Cumulative % | Total | % of Variance | Cumulative % |
| 1 | 21.686 | 63.782 | 63.782 | 10.526 | 30.958 | 30.958 |
| 2 | 2.151 | 6.325 | 70.108 | 5.266 | 15.489 | 46.447 |
| 3 | 1.856 | 5.459 | 75.567 | 4.491 | 13.210 | 59.658 |
| 4 | 1.214 | 3.570 | 79.137 | 2.613 | 7.686 | 67.344 |
| 5 | .966 | 2.841 | 81.978 | 2.355 | 6.926 | 74.270 |
| 6 | .720 | 2.118 | 84.096 | 2.070 | 6.089 | 80.359 |
| 7 | .665 | 1.957 | 86.053 | 1.361 | 4.002 | 84.361 |
| 8 | .562 | 1.652 | 87.705 | 1.137 | 3.343 | 87.705 |
| 9 | .479 | 1.407 | 89.112 |  |  |  |
| 10 | .376 | 1.107 | 90.219 |  |  |  |
| 11 | .356 | 1.048 | 91.267 |  |  |  |
| 12 | .286 | .842 | 92.109 |  |  |  |
| 13 | .281 | .826 | 92.935 |  |  |  |
| 14 | .269 | .791 | 93.726 |  |  |  |
| 15 | .243 | .714 | 94.440 |  |  |  |
| 16 | .222 | .654 | 95.094 |  |  |  |
| 17 | .179 | .526 | 95.619 |  |  |  |
| 18 | .172 | .505 | 96.124 |  |  |  |
| 19 | .166 | .487 | 96.611 |  |  |  |
| 20 | .144 | .424 | 97.035 |  |  |  |
| 21 | .140 | .411 | 97.446 |  |  |  |
| 22 | .134 | .393 | 97.839 |  |  |  |
| 23 | .114 | .336 | 98.176 |  |  |  |
| 24 | .103 | .304 | 98.479 |  |  |  |
| 25 | .089 | .263 | 98.742 |  |  |  |
| 26 | .071 | .210 | 98.952 |  |  |  |
| 27 | .067 | .196 | 99.148 |  |  |  |
| 28 | .062 | .181 | 99.329 |  |  |  |
| 29 | .056 | .166 | 99.495 |  |  |  |
| 30 | .046 | .136 | 99.631 |  |  |  |
| 31 | .043 | .125 | 99.756 |  |  |  |
| 32 | .037 | .108 | 99.864 |  |  |  |
| 33 | .025 | .074 | 99.938 |  |  |  |
| 34 | .021 | .062 | 100.000 |  |  |  |
| Extraction Method: Principal Component Analysis. | | | | | | |
