# Supplementary material for: Validation and the associated factors of the Malay version of systemic lupus erythematosus-specific health-related quality of life questionnaires (SLEQoL and LupusQoL)
Source: PLoS One. 2023 May 15;18(5):e0285461. doi: 10.1371/journal.pone.0285461 (PMC10184909; doi:10.1371/journal.pone.0285461)
Supplement: S5 Table — (DOCX) [file pone.0285461.s007.docx]

Table S5. Exploratory Factor Analysis of 40 items in M-SLEQoL (values below 0.4 are suppressed)

| Rotated Component Matrix^a^ | | | | | | |
| --- | --- | --- | --- | --- | --- | --- |
| Domain/Item | Component | | | | | |
|  | 1 | 2 | 3 | 4 | 5 | 6 |
| Physical function (PF) |  |  |  |  |  |  |
| PF1 |  | .846 |  |  |  |  |
| PF2 |  | .794 |  |  |  |  |
| PF3 |  | .863 |  |  |  |  |
| PF4 |  | .726 | .446 |  |  |  |
| PF5 |  | .841 |  |  |  |  |
| PF6 |  | .470 | .549 |  |  |  |
| Activities (ACTV) |  |  |  |  |  |  |
| ACTV1 |  |  | .698 |  |  |  |
| ACVT2 |  |  | .700 | .435 |  |  |
| ACVT3 |  |  | .654 | .427 |  |  |
| ACTV4 |  | .564 | .536 |  |  |  |
| ACTV5 |  |  | .760 |  |  |  |
| ACTV6 |  |  | .596 |  |  |  |
| ACVT7 |  |  | .687 |  |  |  |
| ACVT8 |  |  | .427 |  | .495 |  |
| ACTV9 |  |  |  |  | .487 |  |
| Symptoms (SYMP) |  |  |  |  |  |  |
| SYMP1 |  |  |  |  | .607 |  |
| SYMP2 |  | .497 |  |  |  |  |
| SYMP3 |  | .420 |  | .522 |  |  |
| SYMP4 |  |  |  | .514 | .456 |  |
| SYMP5 |  |  |  |  | .757 |  |
| SYMP6 |  |  |  |  | .635 |  |
| SYMP7 |  |  |  |  | .726 |  |
| SYMP8 |  |  |  |  | .473 |  |
| Treatment (TR) |  |  |  |  |  |  |
| TR1 |  |  |  | .435 |  | .439 |
| TR2 |  |  |  | .498 |  |  |
| TR3 |  |  |  | .575 |  |  |
| TR4 |  |  |  | .470 |  |  |
| Mood (MOOD) |  |  |  |  |  |  |
| MOOD1 | .438 |  |  | .528 |  |  |
| MOOD2 | .601 |  |  | .458 |  |  |
| MOOD3 | .618 |  |  | .453 |  |  |
| MOOD4 | .619 |  |  | .533 |  |  |
| Self-image (IMAGE) |  |  |  |  |  |  |
| IMAGE1 | .758 |  |  |  |  |  |
| IMAGE2 | .435 |  |  |  |  |  |
| IMAGE3 | .844 |  |  |  |  |  |
| IMAGE4 | .814 |  |  |  |  |  |
| IMAGE5 | .769 |  |  |  |  |  |
| IMAGE6 | .705 |  |  |  |  |  |
| IMAGE7 | .732 |  |  |  |  |  |
| IMAGE8 | .761 |  |  |  |  |  |
| IMAGE9 |  |  |  |  |  | .762 |

The factors were extracted by principal components analysis and varimax-rotated with Kaiser normalization.
