# Supplementary material for: Validation and the associated factors of the Malay version of systemic lupus erythematosus-specific health-related quality of life questionnaires (SLEQoL and LupusQoL)
Source: PLoS One. 2023 May 15;18(5):e0285461. doi: 10.1371/journal.pone.0285461 (PMC10184909; doi:10.1371/journal.pone.0285461)
Supplement: S6 Table — (DOCX) [file pone.0285461.s008.docx]

Table S6. Exploratory Factor Analysis of the 34 items in M-LupusQoL (values below 0.4 are suppressed)

| **Rotated Component Matrix^a^** | | | | | | | | |
| --- | --- | --- | --- | --- | --- | --- | --- | --- |
| Domain/ Item | Component | | | | | | | |
|  | 1 | 2 | 3 | 4 | 5 | 6 | 7 | 8 |
| Physical Function (PF) | | | | | | | | |
| PF1 | .536 |  |  |  |  |  |  | .663 |
| PF2 | .830 |  |  |  |  |  |  |  |
| PF3 | .873 |  |  |  |  |  |  |  |
| PF4 | .865 |  |  |  |  |  |  |  |
| PF5 | .761 |  |  |  |  |  |  |  |
| 6PF6 | .803 |  |  |  |  |  |  |  |
| PF7 | .696 |  |  |  | .413 |  |  |  |
| PF8 | .584 | .495 |  |  |  |  |  |  |
| Pain (PAIN) | | | | | | | | |
| PAIN1 | .575 |  | .496 |  |  |  |  |  |
| PAIN2 | .629 |  |  |  |  |  |  |  |
| PAIN3 | .802 |  |  |  |  |  |  |  |
| Planning (PLAN) | | | | | | | | |
| PLAN1 | .763 |  |  |  |  |  |  |  |
| PLAN2 | .763 |  |  |  |  |  |  |  |
| PLAN3 | .748 |  |  |  |  |  |  |  |
| Inter-relationship (INTRELL) | | | | | | | | |
| INTREL1 |  |  |  |  |  | .971 |  |  |
| INTREL2 |  |  |  |  |  | .969 |  |  |
| Burden (BURDEN) | | | | | | | | |
| BURDEN1 |  |  | .768 |  |  |  |  |  |
| BURDEN2 |  |  | .822 |  |  |  |  |  |
| BURDEN3 |  |  | .754 |  |  |  |  |  |
| Emotion (EMO) |  |  |  |  |  |  |  |  |
| EMO1 | .550 | .561 |  |  |  |  |  |  |
| EMO2 | .595 | .567 |  |  |  |  |  |  |
| EMO3 | .405 | .714 |  |  |  |  |  |  |
| 4EMO |  | .679 | .465 |  |  |  |  |  |
| EMO5 |  | .696 | .451 |  |  |  |  |  |
| EMO6 | .424 | .583 |  |  |  |  |  |  |
| Image (IMAGE) |  |  |  |  |  |  |  |  |
| IMAGE1 |  | .558 |  | .493 |  |  |  |  |
| IMAGE2 | .475 | .490 |  | .553 |  |  |  |  |
| IMAGE3 | .601 |  |  | .515 |  |  |  |  |
| IMAGE4 |  |  |  |  |  |  | .723 |  |
| IMAGE5 |  |  |  | .859 |  |  |  |  |
| Fatigue (FATIGUE) |  |  |  |  |  |  |  |  |
| FATIGUE1 | .413 | .449 |  |  | .480 |  |  |  |
| FATIGUE2 |  |  |  |  | .681 |  |  |  |
| FATIGUE3 |  |  | .437 |  |  |  | .442 | .407 |
| FATIGUE4 | .405 | .504 |  |  | .471 |  |  |  |

The factors were extracted by principal components analysis and varimax-rotated with Kaiser normalization.
