# Supplementary material for: Validation and the associated factors of the Malay version of systemic lupus erythematosus-specific health-related quality of life questionnaires (SLEQoL and LupusQoL)
Source: PLoS One. 2023 May 15;18(5):e0285461. doi: 10.1371/journal.pone.0285461 (PMC10184909; doi:10.1371/journal.pone.0285461)
Supplement: S1 Appendix — (DOC) [file pone.0285461.s009.doc]

SYSTEMIC LUPUS ERYTHEMATOSUS

KAJISELIDIK KUALITI KEHIDUPAN

Bil: ………… Tarikh: (hb/ bln/ thn) …/…/…..

Bahasa: Melayu

Terimakasih kerana melengkapkan borang kaji-selidik ini supaya kami dapat mengetahui dengan lebih lanjut mengenai masalah harian yang dihadapi pesakit-pesakit SLE. Ini akan membantu kami memahami pesakit SLE dengan lebih baik agar dapat mencari cara untuk memperbaiki rawatan SLE secara menyeluruh

Untuk setiap perkara, sila tandakan pada satu bulatan yang paling mengambarkan kepentingannya dalam kehidupan anda, seperti ini **Ø**

Sila jangan meminta bantuan orang lain untuk menjawab soalan kerana andalah orang yang paling mengetahui tentang penyakit anda dan kesannya keatas diri anda. Tiada jawapan yang salah atau betul.

Sila gunakan skala yang diberikan untuk menjawab soalan-soalan yang berikut:

1= langsung tidak susah 2=hampir tidak susah *(tidak susah)* 3= sedikit susah 4=sederhana susah 5=agak susah 6= sangat susah 7 = teramat susah

Dalam 1 minggu yang lalu, apakah tahap kesusahan untuk melakukan perkara-perkara yang berikut akibat penyakit SLE anda:

1 2 3 4 5 6 7

1. Berjalan diluar rumah di tanah yang rata O O O O O O O

2. Membeli belah O O O O O O O

3. Membuka dan menutup kepala paip air atau faucet O O O O O O O

4. Pergi ke gedung membeli belah (kedai runcit) O O O O O O O

5. Mandi dan mengeringkan badan sendiri O O O O O O O

6. Berjalan 3 kilometer ( 2 batu) O O O O O O O

Sila gunakan skala yang berikut untuk menjawab soalan-soalan yang berikut:

1= tidak terganggu langsung 2= hampir tidak terganggu *(tidak terganggu)* 3=sedikit terganggu 4= gangguan yang sederhana 5= agak terganggu 6=sangat terganggu 7= terlalu terganggu

Dalam 1 minggu yang lalu, sejauhmanakah aktiviti sosial atau pekerjaan anda terganggu akibat penyakit SLE anda?

1 2 3 4 5 6 7

7. Prestasi tempat kerja dan sekolah O O O O O O O

8. Menganggu kerjaya atau pendidikan anda O O O O O O O

9. Tidakhadir ke tempat kerja atau sekolah O O O O O O O

10. Hubungan dengan kawan dan saudara-mara O O O O O O O

11. Mengambil bahagian dalam sukan O O O O O O O

12. Hubungan sex O O O O O O O

13. Mengambil bahagian dalam aktiviti- aktiviti sosial O O O O O O O

14. Tidak dapat keluar di bawah matahari O O O O O O O

15. Memperoleh / mendapat kurang duit

kerana saya ada SLE O O O O O O O

Dalam 1 minggu yang lalu, sejauhmanakah anda telah terganggu oleh setiap simptom-simptom ini akibat penyakit SLE anda?:

1 2 3 4 5 6 7

16. Kurang daya ingatan O O O O O O O

17. Hilang selera makan O O O O O O O

18. Sangat lesu O O O O O O O

19. Kurang daya tumpuan O O O O O O O

20. Gatal kulit O O O O O O O

21. Pedih dalam mulut O O O O O O O

22. Pedih, atau sakit kulit O O O O O O O

23. Sakit dan bengkak sendi O O O O O O O

Dalam 1 minggu yang lalu, sejauhmanakah anda telah terganggu oleh setiap masalah berkaitan dengan rawatan perubatan akibat penyakit SLE anda?:

1 2 3 4 5 6 7

24. Takut pada jarum O O O O O O O

25. Had dalam makanan O O O O O O O

26. Kesulitan mengambil ubat setiap hari O O O O O O O

27. Kesulitan untuk kerap ke-klinik O O O O O O O

Sila gunakan skala yang diberikan untuk menjawab soalan-soalan yang seterusnya:

1=tiada langsung 2=hampir tiada *(tiada)* 3=sedikit kerap 4= sederhana kerap 5= agak kerap 6= sangat kerap 7 = terlalu kerap

Dalam 1 minggu yang lalu, berapa kerapkah anda diganggu oleh emosi-emosi berikut akibat penyakit SLE anda?

1 2 3 4 5 6 7

28. Perasaan seolah-olah diperhatikan O O O O O O O

29. Perasan sedih atau murung O O O O O O O

30. Tekanan perasaan O O O O O O O

31. Kebimbangan O O O O O O O

Dalam 1 minggu yang lalu, berapa kerapkah anda terganggu oleh perasaan –perasaan berikut yang akibat penyakit SLE anda?:

1 2 3 4 5 6 7

32. Saya harap orang lain tidak tahu bahawa saya ada SLE O O O O O O O

33. Di persendakan oleh kawan-kawan dan rakan sekerja O O O O O O O

34. Rasa rendah diri O O O O O O O

35. Rasa malu akan SLE saya O O O O O O O

36. Bimbang bebanan kewangan keatas keluarga saya O O O O O O O

37. Bimbang ubat tidak berkesan O O O O O O O

38. Bimbang kesan sampingan ubat O O O O O O O

39. Takut menerima berita buruk daripada doktor O O O O O O O

40. Lebih minum arak atau merokok O O O O O O O

41.Sekiranya anda pernah menjawab borang kaji selidik ini, jawab soalan berikut:

Sejak kali terakhir anda melengkapkan kaji selidik ini, adakah terdapat sebarang perubahan pada keseluruhan kualiti kehidupan anda yang berkaitan dengan SLE anda? (sila tandakan satu)

-7 Terlalu lebih teruk …….

-6 Sangat lebih teruk……

-5 agak lebih teruk……

-4 sederhana lebih teruk……

-3 lebih teruk……

-2 sedikit lebih teruk……

-1 seakan sama ,hampir tidak teruk langsung……

0 tiada perubahan……

1 seakan sama hampir tiada lebih baik langsung……

2 sedikit lebih baik……

3 lebih baik……

4 sederhana lebih baik……

5 agak lebih baik……

6 sangat lebih baik……

7 terlalu lebih baik……
