# Supplementary material for: Validation and the associated factors of the Malay version of systemic lupus erythematosus-specific health-related quality of life questionnaires (SLEQoL and LupusQoL)
Source: PLoS One. 2023 May 15;18(5):e0285461. doi: 10.1371/journal.pone.0285461 (PMC10184909; doi:10.1371/journal.pone.0285461)
Supplement: S2 Appendix — (DOCX) [file pone.0285461.s010.docx]

Appendix 2- LupusQoL Malay

| **Soal Selidik LupusQoL©** | |
| --- | --- |
| Soal selidik berikut direka untuk mengetahui bagaimana SLE menjejaskan kehidupan anda. **Baca** setiap pernyataan kemudian pilih jawapan yang **paling hampir dengan bagaimana anda rasa**. Sila cuba jawab semua soalan sejujur yang boleh. | |
| **Sekerap mana dalam masa 4 minggu yang lalu** | |
| 1. Disebabkan oleh Lupus saya, saya memerlukan pertolongan untuk | 1 Setiap masa |
| melakukan kerja fizikal yang berat seperti menggali taman, mengecat | 2 Kebanyakan masa |
| dan/atau menghias, mengalih perabot | 3 Agakbanyak masa |
|  | 4 Sekali-sekala |
|  | 5 Tidakpernah |
| 2. Disebabkan oleh Lupus saya, saya memerlukan pertolongan untuk | 1 Setiap masa |
| melakukan kerja-kerja fizikal yang sederhana berat seperti menyapu, | 2 Kebanyakan masa |
| menggosok baju, membeli-belah, membersih bilik air | 3 Agakbanyak masa |
|  | 4 Sekali-sekala |
|  | 5 Tidakpernah |
| 3. Disebabkan oleh Lupus saya, saya memerlukan pertolongan untuk melakukan kerja-kerja fizikal yang ringan seperti memasak /menyediakan hidangan makan, membuka balang, mengelap habuk, menyikat rambut atau menguruskan kebersihan diri | 1 Setiap masa  2 Kebanyakan masa  3 Agakbanyak masa  4 Sekali-sekala |
|  | 5 Tidakpernah |
| 4. Disebabkan oleh Lupus saya, saya tidak berupaya untuk melakukan | 1 Setiap masa |
| tugas harian seperti pekerjaan saya, penjagaan anak-anak, kerja rumah | 2 Kebanyakan masa |
| sebaik yang saya mahukan | 3 Agakbanyak masa |
|  | 4 Sekali-sekala |
|  | 5 Tidakpernah |
| 5. Disebabkan oleh Lupus saya, saya sukar menaiki tangga | 1 Setiap masa |
|  | 2 Kebanyakan masa |
|  | 3 Agakbanyak masa |
|  | 4 Sekali-sekala |
|  | 5 Tidakpernah |
| 6. Disebabkan oleh Lupus saya, saya kehilangan sedikit kebebasan dan bergantung pada orang lain | 1 Setiap masa  2 Kebanyakan masa |
|  | 3 Agakbanyak masa |
|  | 4 Sekali-sekala |
|  | 5 Tidakpernah |
| 7. Saya harus melakukan perkara pada kadar yang lebih perlahan kerana | 1 Setiap masa |
| Lupus saya | 2 Kebanyakan masa |
|  | 3 Agakbanyak masa |
|  | 4 Sekali-sekala |
|  | 5 Tidakpernah |
| 8. Disebabkan oleh Lupus saya, corak tidur saya terganggu | 1 Setiap masa |
|  | 2 Kebanyakan masa |
|  | 3 Agakbanyak masa |
|  | 4 Sekali-sekala |
|  | 5 Tidakpernah |
| 9. Saya terhalang daripada melakukan aktiviti dengan cara yang saya mahu akibat kesakitan kerana Lupus | 1 Setiap masa  2 Kebanyakan masa |
|  | 3 Agakbanyak masa |
|  | 4 Sekali-sekala |
|  | 5 Tidakpernah |

| **Soal Selidik LupusQoL©** *(bersambung)* | |
| --- | --- |
| **Sekerap mana dalam masa 4 minggu yang lalu** | |
| 10. Disebabkan oleh Lupus saya, kesakitan yang saya alami mengganggu | 1 Setiap masa |
| mutu tidur saya | 2 Kebanyakan masa |
|  | 3 Agakbanyak masa |
|  | 4 Sekali-sekala |
|  | 5 Tidakpernah |
| 11. Kesakitan akibat Lupus saya amat teruk sehingga ia mengehadkan kebolehgerakan saya | 1 Setiap masa  2 Kebanyakan masa |
|  | 3 Agakbanyak masa |
|  | 4 Sekali-sekala |
|  | 5 Tidakpernah |
| 12. Disebabkan oleh Lupus saya, saya mengelak daripada merancang untuk | 1 Setiap masa |
| menghadiri acara pada masa depan | 2 Kebanyakan masa |
|  | 3 Agakbanyak masa |
|  | 4 Sekali-sekala |
|  | 5 Tidakpernah |
| 13. Kerana ketidaktentuan Lupus saya, saya tidak boleh menguruskan | 1 Setiap masa |
| kehidupan saya dengan cekap | 2 Kebanyakan masa |
|  | 3 Agakbanyak masa |
|  | 4 Sekali-sekala |
|  | 5 Tidakpernah |
| 14. Lupus saya berubah dari hari ke hari membuatkan saya sukar untuk terikat kepada pengaturan sosial | 1 Setiap masa  2 Kebanyakan masa |
|  | 3 Agakbanyak masa |
|  | 4 Sekali-sekala |
|  | 5 Tidakpernah |
| 15. Disebabkan oleh kesakitan yang saya alami akibat Lupus, saya kurang | 1 Setiap masa |
| berminat dengan hubungan seksual | 2 Kebanyakan masa |
|  | 3 Agakbanyak masa |
|  | 4 Sekali-sekala |
|  | 5 Tidakpernah |
|  | 793 Tidakberkenaan |
| 16. Disebabkan oleh Lupus saya, saya tidak berminat dengan seks | 1 Setiap masa |
|  | 2 Kebanyakan masa |
|  | 3 Agakbanyak masa |
|  | 4 Sekali-sekala |
|  | 5 Tidakpernah |
|  | 793 Tidakberkenaan |
| 17. Saya bimbang Lupus saya memberi tekanan kepada mereka yang rapat | 1 Setiap masa |
| dengan saya | 2 Kebanyakan masa |
|  | 3 Agakbanyak masa |
|  | 4 Sekali-sekala |
|  | 5 Tidakpernah |
| 18. Disebabkan oleh Lupus saya, saya bimbang bahawa saya menyebabkan mereka yang rapat dengan saya risau | 1 Setiap masa  2 Kebanyakan masa |
|  | 3 Agakbanyak masa |
|  | 4 Sekali-sekala |
|  | 5 Tidakpernah |

| **Soal Selidik LupusQoL©** *(bersambung)* | |
| --- | --- |
| **Sekerap mana dalam masa 4 minggu yang lalu** | |
| 19. Disebabkan oleh Lupus saya, saya berasa bahawa saya merupakan beban kepada rakan dan/atau keluarga | 1 Setiap masa  2 Kebanyakan masa |
|  | 3 Agakbanyak masa |
|  | 4 Sekali-sekala |
|  | 5 Tidakpernah |
| **Dalam masa 4 minggu lalu saya dapati Lupus saya membuatkan saya** | |
| 20. Geram | 1 Setiap masa |
|  | 2 Kebanyakan masa |
|  | 3 Agakbanyak masa |
|  | 4 Sekali-sekala |
|  | 5 Tidakpernah |
| 21. Sangat bosan tiada apa yang boleh menceriakan saya | 1 Setiap masa |
|  | 2 Kebanyakan masa |
|  | 3 Agakbanyak masa |
|  | 4 Sekali-sekala |
|  | 5 Tidakpernah |
| 22. Sedih | 1 Setiap masa |
|  | 2 Kebanyakan masa |
|  | 3 Agakbanyak masa |
|  | 4 Sekali-sekala |
|  | 5 Tidakpernah |
| 23. Khuatir | 1 Setiap masa |
|  | 2 Kebanyakan masa |
|  | 3 Agakbanyak masa |
|  | 4 Sekali-sekala |
|  | 5 Tidakpernah |
| 24. Risau | 1 Setiap masa |
|  | 2 Kebanyakan masa |
|  | 3 Agakbanyak masa |
|  | 4 Sekali-sekala |
|  | 5 Tidakpernah |
| 25. Kurang keyakinan diri | 1 Setiap masa |
|  | 2 Kebanyakan masa |
|  | 3 Agakbanyak masa |
|  | 4 Sekali-sekala |
|  | 5 Tidakpernah |
| **Sekerap mana dalam masa 4 minggu yang lalu** | |
| 26. Penampilan fizikal saya yang disebabkan oleh Lupus mengganggu keseronokan hidup saya | 1 Setiap masa  2 Kebanyakan masa |
|  | 3 Agakbanyak masa |
|  | 4 Sekali-sekala |
|  | 5 Tidakpernah |

| **Soal Selidik LupusQoL©** *(bersambung)* | |
| --- | --- |
| **Sekerap mana dalam masa 4 minggu yang lalu** | |
| 27. Disebabkan oleh Lupus saya, penampilan saya (contohnya, ruam, pertambahan/penurunan berat badan) membuatkan saya mengelak daripada situasi sosial | 1 Setiap masa  2 Kebanyakan masa  3 Agakbanyak masa  4 Sekali-sekala  5 Tidakpernah  793 Tidakberkenaan |
| 28. Ruam kulit yang berkait dengan Lupus membuatkan saya berasa kurang menarik | 1 Setiap masa  2 Kebanyakan masa  3 Agakbanyak masa  4 Sekali-sekala  5 Tidakpernah  793 Tidakberkenaan |
| 29. Keguguran rambut yang saya alami akibat Lupus saya membuat saya berasa kurang menarik | 1 Setiap masa  2 Kebanyakan masa  3 Agakbanyak masa  4 Sekali-sekala  5 Tidakpernah  793 Tidakberkenaan |
| 30. Pertambahan berat badan yang saya alami akibat rawatan Lupus saya membuat saya berasa kurang menarik | 1 Setiap masa  2 Kebanyakan masa  3 Agakbanyak masa  4 Sekali-sekala  5 Tidakpernah  793 Tidakberkenaan |
| 31. Disebabkan oleh Lupus saya, saya tidak boleh menumpukan perhatian untuk tempoh masa yang panjang | 1 Setiap masa  2 Kebanyakan masa  3 Agakbanyak masa  4 Sekali-sekala  5 Tidakpernah |
| 32. Disebabkan oleh Lupus saya, saya berasa letih dan lembab | 1 Setiap masa |
|  | 2 Kebanyakan masa |
|  | 3 Agakbanyak masa |
|  | 4 Sekali-sekala |
|  | 5 Tidakpernah |
| 33. Disebabkan oleh Lupus saya, saya perlu tidur awal | 1 Setiap masa |
|  | 2 Kebanyakan masa |
|  | 3 Agakbanyak masa |
|  | 4 Sekali-sekala |
|  | 5 Tidakpernah |
| 34. Disebabkan oleh Lupus saya, saya selalu keletihan pada waktu pagi | 1 Setiap masa |
|  | 2 Kebanyakan masa |
|  | 3 Agakbanyak masa |
|  | 4 Sekali-sekala |
|  | 5 Tidakpernah |
| **Sila semak untuk memastikan anda telah menjawab semua soalan Terima Kasih kerana sudi menyertai soal selidik ini.**  **© 2006. UniversityofCentralLancashire&EastLancashireHospitals NHS Trust. Hak cipta terpelihara. Tidak boleh diterbitkan semula seluruh atau sebahagiannya tanpa kebenaran pemegang hak cipta.** | |
